# Long-term environmental enrichment is associated with better fornix microstructure in older adults

**DOI:** 10.3389/fnagi.2023.1170879

**Published:** 2023-08-28

**Authors:** Olga M. Klimecki, Maxie Liebscher, Malo Gaubert, Dayana Hayek, Alexis Zarucha, Martin Dyrba, Claudia Bartels, Katharina Buerger, Michaela Butryn, Peter Dechent, Laura Dobisch, Michael Ewers, Klaus Fliessbach, Silka Dawn Freiesleben, Wenzel Glanz, Stefan Hetzer, Daniel Janowitz, Ingo Kilimann, Luca Kleineidam, Christoph Laske, Franziska Maier, Matthias H. Munk, Robert Perneczky, Oliver Peters, Josef Priller, Boris-Stephan Rauchmann, Nina Roy, Klaus Scheffler, Anja Schneider, Eike Jakob Spruth, Annika Spottke, Stefan J. Teipel, Jens Wiltfang, Steffen Wolfsgruber, Renat Yakupov, Emrah Düzel, Frank Jessen, Michael Wagner, Sandra Roeske, Miranka Wirth, the DELCODE study group

**Affiliations:** ^1^German Center for Neurodegenerative Diseases (DZNE), Dresden, Germany; ^2^Department of Neuroradiology, Rennes University Hospital Centre Hospitalier Universitaire (CHU), Rennes, France; ^3^German Center for Neurodegenerative Diseases (DZNE), Magdeburg, Germany; ^4^German Center for Neurodegenerative Diseases (DZNE), Rostock, Germany; ^5^Department of Psychiatry and Psychotherapy, University Medical Center Goettingen, University of Goettingen, Göttingen, Germany; ^6^German Center for Neurodegenerative Diseases (DZNE), Munich, Germany; ^7^Institute for Stroke and Dementia Research, University Hospital, Ludwig-Maximilians-Universität (LMU) Munich, Munich, Germany; ^8^Magnetic Resonance (MR)-Research in Neurosciences, Department of Cognitive Neurology, Georg-August-University Goettingen, Göttingen, Germany; ^9^German Center for Neurodegenerative Diseases (DZNE), Bonn, Germany; ^10^Department of Neurodegenerative Disease and Geriatric Psychiatry, University of Bonn Medical Center, Bonn, Germany; ^11^Department of Psychiatry and Neurosciences, Charité–Universitätsmedizin Berlin, Berlin, Germany; ^12^German Center for Neurodegenerative Diseases (DZNE), Berlin, Germany; ^13^Berlin Center for Advanced Neuroimaging, Charité–Universitätsmedizin Berlin, Berlin, Germany; ^14^Department of Psychosomatic Medicine, Rostock University Medical Center, Rostock, Germany; ^15^German Center for Neurodegenerative Diseases (DZNE), Tübingen, Germany; ^16^Section for Dementia Research, Department of Psychiatry and Psychotherapy, Hertie Institute for Clinical Brain Research, University of Tübingen, Tübingen, Germany; ^17^Department of Psychiatry, Medical Faculty, University of Cologne, Cologne, Germany; ^18^Department of Psychiatry and Psychotherapy, University of Tübingen, Tübingen, Germany; ^19^Department of Psychiatry and Psychotherapy, University Hospital, Ludwig-Maximilians-Universität (LMU) Munich, Munich, Germany; ^20^Munich Cluster for Systems Neurology (SyNergy), Munich, Germany; ^21^Ageing Epidemiology Research Unit (AGE), School of Public Health, Imperial College London, London, United Kingdom; ^22^Department of Psychiatry and Psychotherapy, Charité–Universitätsmedizin Berlin, Berlin, Germany; ^23^Department of Psychiatry and Psychotherapy, School of Medicine, Technical University of Munich, Munich, Germany; ^24^University of Edinburgh and United Kingdom Dementia Research Institute (UK DRI), Edinburgh, United Kingdom; ^25^Department for Biomedical Magnetic Resonance, University of Tübingen, Tübingen, Germany; ^26^Department of Neurology, University of Bonn, Bonn, Germany; ^27^German Center for Neurodegenerative Diseases (DZNE), Göttingen, Germany; ^28^Neurosciences and Signaling Group, Department of Medical Sciences, Institute of Biomedicine (iBiMED), University of Aveiro, Aveiro, Portugal; ^29^Institute of Cognitive Neurology and Dementia Research (IKND), Otto-von-Guericke University, Magdeburg, Germany; ^30^Excellence Cluster on Cellular Stress Responses in Aging-Associated Diseases, University of Cologne, Cologne, Germany

**Keywords:** multimodal leisure activities, brain reserve, brain plasticity, memory, prevention, Alzheimer’s disease

## Abstract

**Background:**

Sustained environmental enrichment (EE) through a variety of leisure activities may decrease the risk of developing Alzheimer’s disease. This cross-sectional cohort study investigated the association between long-term EE in young adulthood through middle life and microstructure of fiber tracts associated with the memory system in older adults.

**Methods:**

*N* = 201 cognitively unimpaired participants (≥ 60 years of age) from the DZNE-Longitudinal Cognitive Impairment and Dementia Study (DELCODE) baseline cohort were included. Two groups of participants with higher (*n* = 104) or lower (*n* = 97) long-term EE were identified, using the self-reported frequency of diverse physical, intellectual, and social leisure activities between the ages 13 to 65. White matter (WM) microstructure was measured by fractional anisotropy (FA) and mean diffusivity (MD) in the fornix, uncinate fasciculus, and parahippocampal cingulum using diffusion tensor imaging. Long-term EE groups (lower/higher) were compared with adjustment for potential confounders, such as education, crystallized intelligence, and socio-economic status.

**Results:**

Reported participation in higher long-term EE was associated with greater fornix microstructure, as indicated by higher FA (standardized β = 0.117, *p* = 0.033) and lower MD (β = −0.147, *p* = 0.015). Greater fornix microstructure was indirectly associated (FA: unstandardized *B* = 0.619, *p* = 0.038; MD: *B* = −0.035, *p* = 0.026) with better memory function through higher long-term EE. No significant effects were found for the other WM tracts.

**Conclusion:**

Our findings suggest that sustained participation in a greater variety of leisure activities relates to preserved WM microstructure in the memory system in older adults. This could be facilitated by the multimodal stimulation associated with the engagement in a physically, intellectually, and socially enriched lifestyle. Longitudinal studies will be needed to support this assumption.

## 1. Introduction

The worldwide prevalence of AD is expected to reach 153 million cases by 2050 (Nichols et al., 2022). Progressive deterioration of the memory system and associated function is a core symptom of Alzheimer’s disease (AD) (e.g., Sperling et al., 2011). AD-related memory loss and clinical progression are closely linked to accelerated brain atrophy in medio-temporal brain structures and their white matter (WM) connections, including the fornix tract as the major pathway of the hippocampus (Mielke et al., 2012). In the absence of a cure for AD, tremendous effort has been placed on developing treatment strategies focusing on the prevention of AD through protective lifestyle factors comprising physical, intellectual and social activities (Livingston et al., 2020). Participation in a greater variety of enriching leisure activities may have a particularly positive impact on brain health, including the WM microstructure of the memory system; however, these neurophysiological relations remain to be determined in older adults (OA).

Existing studies have indicated that sustained multimodal environmental enrichment (EE), combining motor, cognitive, sensory, and social stimulation, has far-reaching benefits for the brain. In animal models, groups of rats or mice exposed to EE show greater benefits compared to groups of animals with low EE (i.e., standard housing), particularly in the memory system including hippocampal structure and function (Fabel et al., 2009; Kempermann et al., 2010; Kempermann, 2019; Manno et al., 2022). In humans, multimodal activities such as regular musical instrument playing or dancing have been associated with a reduced risk of developing dementia (Verghese et al., 2003). Likewise, participating in a diversity of enriching leisure activities, including physical and cognitive activities, could be beneficial in preserving or enhancing the brain and cognition. This has been suggested by various cross-sectional (Wirth et al., 2014; Porat et al., 2016; Chan et al., 2018), longitudinal (Karp et al., 2006), and interventional (Theill et al., 2013; Ngandu et al., 2015; Köbe et al., 2016) studies as well as systematic reviews and meta-analyses (Lauenroth et al., 2016; Gavelin et al., 2021; Torre and Temprado, 2022). A study of particular interest conducted in OA over 80 years showed that 3 year changes in WM microstructure in the corticospinal tract accounted for the association between changes in self-reported leisure activities and changes in perceptual speed (Köhncke et al., 2016).

Despite these insights, the potential brain correlates associated with sustained or long-term EE in the memory system of OA remain unclear. Age-related alterations in WM microstructure, as defined by lower fractional anisotropy (FA) and higher mean diffusivity (MD) measured using diffusion-weighted imaging (DWI), begin and are most severe in the fornix, the uncinate fasciculus, and the parahippocampal cingulum (Teipel et al., 2016; Pichet Binette et al., 2021). Disruption of these critical fiber tracts has been associated with lower episodic memory functioning (Huang et al., 2007; Fellgiebel et al., 2008; Sexton et al., 2010; Metzler-Baddeley et al., 2011; Hayek et al., 2020) and cognitive worsening over time (Mielke et al., 2012; Zhuang et al., 2012; Fletcher et al., 2013) in OA. Conversely, successful preservation or adaptation of WM microstructure (as indicated by higher FA and lower MD) through enriching leisure activities could help protect the memory system in late life and strengthen brain reserve (Teipel et al., 2016; Xu et al., 2019; Luo et al., 2020). Evidence syntheses regarding lifestyle activities, such as physical or cognitive activities, and their association with WM microstructure in OA have provided inconclusive results (Sexton et al., 2016; Anatürk et al., 2018). However, participation in multimodal activities, including a 6-months piano training and a 6-months dance training, has been associated with a positive impact on fornix microstructure in OA (Burzynska et al., 2017; Jünemann et al., 2022).

Here, we investigated the cross-sectional association between sustained EE in young and middle adulthood and WM microstructure of the memory system in OA from the DZNE-Longitudinal Cognitive Impairment and Dementia (DELCODE) study (Jessen et al., 2018). Long-term EE was measured by the self-reported frequency of participation in a variety of enriching leisure activities, including physical, intellectual and social activities, between the ages 13 and 65. We hypothesized that participants with higher long-term EE would have more favorable microstructure in three WM tracts, namely the fornix, uncinate fasciculus, and parahippocampal cingulum, compared to participants with lower long-term EE. We carefully controlled for other known reserve proxies, such as education, crystallized intelligence and socio-economic status (SES) (Stern, 2009), as done in our previous study in the DELCODE cohort (Böttcher et al., 2022). Lastly, we explored neuroprotective path models (Wirth et al., 2014) conceptualized as brain maintenance and brain reserve models to better understand the interplay among long-term EE, WM microstructure and memory function in the OA.

## 2. Materials and methods

This study was based on cross-sectional baseline data that were obtained from the ongoing multicenter observational DZNE-DELCODE study (Jessen et al., 2018). The DELCODE study was designed according to the ethical principles of the Declaration of Helsinki. Local ethical committees at each participating study site approved the study protocol. All participants gave written informed consent. The DELCODE study was registered at the German Clinical Trials Register (DRKS00007966; date: 2015/05/04).

### 2.1. Participants

Based on our research questions, participants in the cognitively normal range were included from the baseline DELCODE database. Specifically, our sample included older adults, participants with a family history of AD, and participants with subjective cognitive decline to increase sample size and statistical power. Briefly, all participants had fluent German skills and were 60 years of age or older. All participants included in this study had normal cognitive performance, as determined using the Consortium to Establish a Registry of Alzheimer’s Disease (CERAD) battery (Morris et al., 1989). Past or present major psychiatric, medical, or neurological disorders were set as exclusion criteria. In total, the current sample included *N* = 201 participants. The selection flow chart is provided in the Supplementary material (Supplementary Figure 1).

### 2.2. Measurements

#### 2.2.1. Long-term environmental enrichment

Long-term EE was operationalized using items from a German version (LEQ-D, Roeske et al., 2018) of the Lifetime of Experiences Questionnaire (LEQ; Valenzuela and Sachdev, 2007). The LEQ is an established questionnaire that assesses, among others, a wide range of leisure activities. The LEQ has been translated into multiple languages (Ourry et al., 2021) and has been implemented in previous studies on cognitive reserve (Chan et al., 2018; Collins et al., 2021).

To measure EE, we used items of the non-specific subscore of LEQ. The non-specific LEQ items are a validated measure of cognitive, artistic and social engagement across life (Valenzuela and Sachdev, 2007). Indeed, a factor analysis of the original LEQ (Valenzuela and Sachdev, 2007) indicated two main factors: factor one that captures education and occupation (the specific LEQ factor) and factor two that captures social, artistic, and musical activities (the non-specific LEQ). This factor structure was recently replicated in a cross-country validation of the LEQ in four European countries (Ourry et al., 2021). This study confirmed the essential factor structure for the specific and non-specific parts of the LEQ across the sample and for each country (Ourry et al., 2021). As the non-specific LEQ factor is closest to the concept of environmental enrichment from animal research, we use specific items of interest from the non-specific LEQ that capture environmental enrichment it in the current study. In this way, our approach is similar to previous studies (Oltmanns et al., 2017).

More specifically, we selected unspecific items of the LEQ-D questionnaire measuring the frequency of participation in 6 leisure activities on a 6-point Likert scale ranging from 0 (never), 1 (less than 1 time per month), 2 (1 time per month), 3 (2 times per month), 4 (weekly), to 5 (daily). These items are related to (1) social activity (seeing family members), (2) musical activity (playing a musical instrument) (3) artistic activity (e.g., drawing), (4) physical activity, (5) reading (as a proxy for cognitive activity), and (6) speaking an additional language. Participation in these leisure activities was rated retrospectively for three life periods, i.e., young adulthood (13–30 years), mid-life (30–65 years), and late-life (65 years and older). Only items concerning young adulthood and mid-life were included in this study, as some participants were below 65 years of age.

There are several ways to define and analyze long-term EE. From a conceptual point of view, we aimed to compare the “effects” of long-term EE between groups of OA with lower or higher frequency of participation in enriching leisure activities. This group-based comparison (lower vs. higher EE) was inspired by the classical enriched environment paradigm, as established in animal (rat and mice) models (Kempermann, 2019). In the present (human) data, we used a data-driven approach and evaluated long-term EE as a binary group variable defined by median split with lower and higher frequency of participation in the given leisure activities. Similar binary evaluations of leisure activities have been reported by previous studies (Böttcher et al., 2022; Duffner et al., 2022). Briefly, we summed the scores across the 6 leisure activities as assessed by the LEQ per life period with a maximum total score of 30 per life period. The median score of the summed leisure activities was 18 for younger adulthood and 16 for mid-life. Participants with higher EE across both life periods (median and higher) were included in the higher EE group (*n* = 104), while participants with lower EE across both life periods (below median) were included in the lower EE group (*n* = 97).

#### 2.2.2. MRI acquisition and pre-processing

The magnetic resonance imaging (MRI) data were collected in nine participating sites, each equipped with a Siemens 3.0 Tesla MRI scanner (one Prisma, one Skyra, three TimTrio, and four Verio systems). Scanning instructions were standardized across sites and identical MRI protocol acquisition parameters were used. Details on sequence parameters are provided in the Supplementary material.

Diffusion-weighted MRI data were preprocessed with the FMRIB software library (FSL, Version 6.0.4. Jenkinson et al., 2012). The brain extraction tool (bet) was used for skull stripping. Images were corrected for distortions with a gradient-echo field map using the fsl_prepare_fieldmap and epi_reg commands. Then, images were corrected for eddy currents using the command eddy_correct and the first b0 image as a reference volume. After, bvecs were rotated to preserve correct orientation information with the command fsl_rotate_bvecs (see Leemans and Jones, 2009). The command dtifit was used to fit a diffusion tensor model at each voxel and to create the FA and MD maps. For all participants, head movement, defined by the Euclidian distance in millimeters of how much the head was moved, was calculated using the eddy_correct log file (Tromp, 2016).

#### 2.2.3. MRI analysis

To extract FA and MD values from the pre-selected fiber tracts (i.e., fornix, the uncinate fasciculus, and the parahippocampal cingulum), a tract-of-interest (TOI) based approach was chosen, following the procedure described by Antonenko et al. (2016). All TOI masks were extracted based on the 2 mm isotropic resolution Montreal Neurological Institute (MNI) template provided by FSL. Masks for the fornix and uncinate fasciculus were extracted from the probabilistic Juelich histological atlas (Bürgel et al., 2006). The mask for the parahippocampal cingulum was extracted from the non-probabilistic JHU ICBM-DTI-81 atlas (Oishi et al., 2008). Based on previous research (Antonenko et al., 2016) and visual inspection, voxels of probabilistic masks of the fornix were thresholded at 50% probability for inclusion in the mask, the uncinate fasciculus mask was thresholded at 20%. The mask for the parahippocampal cingulum was not probabilistic and, therefore, directly used as provided.

As already provided in the description of section “2.2.2. MRI acquisition and pre-processing,” we transformed the atlas-based tracts-of interest (TOI) from Montreal Neurological Institute and Hospital (MNI) reference space to individual subject native space. The TOI masks were binarized and transformed from standard space into native space to extract diffusion metrics using the statistical parametric mapping toolbox (SPM12, version 7487, Wellcome Trust Centre for Neuroimaging, London, UK)^1^ with the segmentation algorithm of the computational anatomy toolbox (CAT12, version 12.6, rl1450)^2^ implemented in Matlab 2018a (Mathworks, Natwick). The computational anatomy toolbox (CAT12) provides a one-click procedure to perform image segmentation [gray matter, white matter, cerebrospinal fluid (CSF)] and estimation of deformation fields for warping the native space t1-weighted MRI scans to MNI space. The inverse deformation field can be used to warp the images back from MNI space to native subject space. As the t1-weighted scans and diffusion scans were initially coregistered to each other, we could directly apply the deformations to the MNI TOI masks to bring the masks in the respective subject space. Subsequently, the mean FA and MD values per TOI were extracted for each participant. As the fornix tract borders the lateral ventricles, it is important to correct for CSF contamination (Hayek et al., 2020). An FA value below 0.1 and an MD value above 2.5e-03 is likely to reflect CSF rather than brain tissue (Hasan et al., 2014). Therefore, these thresholds were used for all voxels of individually extracted fornix FA and MD masks.

#### 2.2.4. Measurement of additional variables

Co-variates of no interest considered in the present study were age, gender, education, crystalized intelligence, socio-economic status (SES), diagnostic category, and scanner site. Most of these measures are provided by the baseline database of the DELCODE study and were described in our previous study (Böttcher et al., 2022). In brief, educational attainment was assessed in years of education. Vocabulary was used as a proxy for crystallized intelligence, estimated by the total score of the German Mehrfachwahl-Wortschatz-Intelligenztest (MWT, English: Multiple-Choice Vocabulary Intelligence Test, Lehrl, 2005). MWT scores range from 0 to 37 with higher scores corresponding to higher crystallized intelligence. The SES was estimated by the international socio-economic index of occupational information (ISEI) (Ganzeboom et al., 1992). The measure was calculated using the self-reported occupational history assessed by the LEQ-D and established crosswalk procedures. ISEI scores range from 16 to 90 with higher scores corresponding to higher SES. The scanner site was included to account for scanner-related variance in the diffusion tensor imaging (DTI) measures (Brueggen et al., 2019; Finsterwalder et al., 2020).

### 2.3. Statistical analysis

Statistical analysis was conducted in IBM SPSS 23 and RStudio Version 1.4.1103 (R Core Team, 2020). The mediation package (version 4.5.0) was used to construct path models (Tingley et al., 2014). An alpha value of 0.05 was considered statistically significant. As the TOIs were considered as independent from each other, tests were not corrected for multiple comparisons. Statistical assumptions for each analysis were checked following guidelines provided by Field (2013). Plots presented in the results section were plotted in R with the package ggplot2 (version 3.3.5) (Wickham, 2016).

#### 2.3.1. Sample characteristics

Long-term EE groups (lower/higher) were first compared on baseline demographic, behavioral, neuropsychological, and neuroimaging variables. Statistical comparisons were conducted with independent samples *t*-tests for continuous variables and chi-squared (χ^2^) tests for categorical variables.

#### 2.3.2. Analysis of main hypotheses

To assess our main hypothesis for WM microstructure, multiple linear regression models were computed with FA or MD of each TOI (fornix, uncinate fasciculus, and parahippocampal cingulum) as dependent variables and long-term EE (lower/higher) as an independent variable. In all regression models, we accounted for age, gender, education, crystallized intelligence, SES, and diagnostic category. Furthermore, we tested for multicollinearity among all covariates using simple Pearson correlation and VIF (variance inflation factor). In the Pearson correlation, the highest correlation was between the covariates education and SES (*r* = 0.59). According to Field et al. (2012), multicollinearity is only present when the correlation coefficient reaches 0.9 or −0.9. In line with this result, the VIF method also indicated that there is no multicollinearity among the covariates, as the highest values were observed for education (1.67) and SES (1.74), and according to Kutner et al. (2005), VIFs exceeding 10 may indicate multicollinearity. To minimize site-effects, regression models for TOIs included scanner site as a categorical covariate. All categorical covariates were included as dummy coded variables in all models specified above, granted they had more than two levels. For all regression models, the lower long-term EE group was the reference category for long-term EE, female was the reference category for gender, and OA was the reference category for the diagnostic category.

#### 2.3.3. Exploratory path analysis

We conducted exploratory path analyses using the mediation package v.4.5.0 (Tingley et al., 2014) in RStudio version 1.4.1103 (R Core Team, 2020) and adjusted for co-variates of no interest, i.e., age, gender, education, crystallized intelligence, SES, diagnostic category, and scanner location, in a methodological approach similar to Wirth et al. (2014). Path models were computed for fornix FA and MD similar to a previous study (Hayek et al., 2020). One path model, here conceptualized as brain maintenance model, assessed long-term EE as independent variable, fornix FA/MD as the mediator, and memory function as dependent variable. The second (reverse) path model, here conceptualized as brain reserve model, assessed fornix FA/MD as independent variable, long-term EE as mediator, and memory function as dependent variable. We examined the contribution of the indirect (mediator) effect as the outcome of interest, computed by the product of path coefficients, similar to our preceding study (Wirth et al., 2014). Indirect effects were evaluated using bias-corrected 95% confidence intervals (CI) established with 5,000 bootstrap samples. The indirect effect was considered significant if the CI did not include the value zero.

In both path models, we evaluated memory function by the neuropsychological learning and memory (MEM) factor score, in detail described elsewhere (Wolfsgruber et al., 2020). The incorporated neuropsychological tests were provided by Wolfsgruber et al. (2020). In brief, they consist of the following tests: performance in learning and memory (MEM) was assessed using a latent factor score that was previously constructed based on the extensive neuropsychological test battery of the DELCODE cohort (Wolfsgruber et al., 2020). The MEM factor score included the Free and Cued Selective Reminding Test (FCSRT, Grober et al., 2009), the Logical Memory Test of the Wechsler Memory Scale (WMS-IV, Lepach and Petermann, 2012), the incidental learning scale of the Symbol Digit Modalities Test (Smith, 1982), the computerized Face Name Associative Recognition Test (Polcher et al., 2017), the word list learning, recall, and recognition of the ADAS-Cog 13 (Mohs et al., 1997) and a recall task of previously copied figures (analogous to the CERAD battery, Thalmann et al., 2000). For the present purpose, the MEM factor score was z-transformed using the mean and standard deviation of the present sample (*N* = 201).

#### 2.3.4. *Post hoc* group characterization

A *post hoc* comparison was performed to explore relative differences in the frequency of participation across the 6 different leisure activities (as assessed by the LEQ-D) between higher and lower long-term EE groups. We conducted simple regression models with long-term EE (lower/higher) as the independent variable and the respective leisure activity (language, artistic, musical, physical, reading, or social) as the dependent variable. For the present purpose, the effect size measure (standardized regression coefficient) was evaluated, classified as small (0.2–0.5), medium (0.5–0.8) or large (0.8 and larger) effects (Ferguson, 2009).

## 3. Results

### 3.1. Sample characteristics

This study included a total of *N* = 201 participants from the DELCODE study. Of those, *n* = 97 were in the lower long-term EE group and *n* = 104 participants were in the higher long-term EE group. A summary of the sample characteristics at baseline including demographics, behavioral and biological measures is given in Table 1. Participants with higher long-term EE had significantly higher years of education, crystallized intelligence, and SES compared to participants with lower EE. The two groups were comparable in the other baseline characteristics.

**TABLE 1:** Descriptive characterization of the final sample.

	Whole sample (*N* = 201) M (SD)	Lower EE (*n* = 97) M (SD)	Higher EE (*n* = 104) M (SD)	Test statistics	*p*
Age (years)	69.10 (6.28)	69.25 (6.19)	68.97 (6.38)	*t* = 0.311	0.756
Gender female/male (*n*)	97/104	46/51	51/53	χ^2^ = 0.052	0.819
Education (years)	15.00 (2.93)	14.18 (2.71)	15.77 (2.93)	*t* = −4.008	< 0.001***
Crystalized intelligence^a^	32.51 (2.61)	31.91 (2.59)	33.07 (2.52)	*t* = −3.214	0.002**
SES^b^	62.76 (17.06)	58.28 (17.02)	66.95 (16.07)	*t* = −3.714	< 0.001***
Diagnostic category OA/SCD/FH (*n*)	84/90/27	42/41/14	42/49/13	χ^2^ = 0.505	0.777
APOE ε4 allele carrier, *n* (%)^c^	55 (27.8)	28 (29.5)	27 (26.2)	χ^2^ = 0.125	0.724
BMI^d^	25.71 (3.35)	25.83 (3.36)	25.59 (3.35)	*t* = 0.520	0.604
Total intracranial volume (ml)	1,405.628 (200.991)	1,412.816 (213.300)	1,398.923 (189.585)	*t* = 0.487	0.627

*^a^*Multiple-Choice Vocabulary Intelligence Test (MWT).

*^b^*International socio-economic index (ISEI).

*^c^*Measure available in *n* = 198 participants (lower EE: *n* = 95, higher EE: *n* = 103).

*^d^*Measure was calculated using the following formula: weight (kilograms)/height^2^ (meters).

Descriptive data are given if applicable as mean and standard deviation (in parenthesis). For continuous variables, *p*-values correspond to independent *t*-tests with unequal variances with long-term EE (levels lower/higher) as the independent variable. For categorical variables, the chi-square statistic was used to compare the distribution.

***p < 0.001, **p < 0.01.

M, mean; SD, standard deviation. Key: BMI, body mass index; OA, older adults; FH, participants with a family history of AD; SCD, subjective cognitive decline; SES, socioeconomic status; MMSE, mini-mental state examination, GDS, geriatric depression scale.

### 3.2. Association between long-term EE and WM microstructure

**FIGURE 1:**
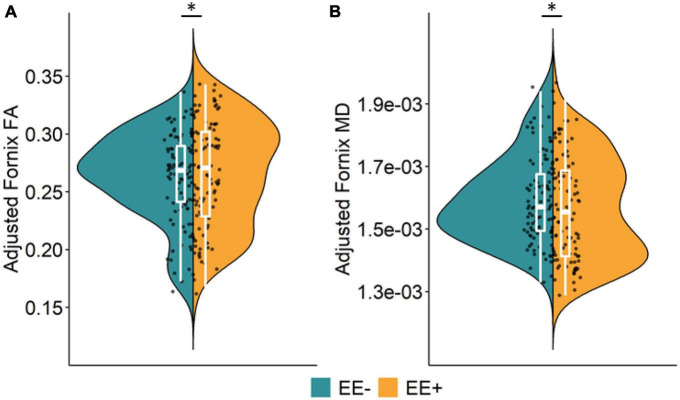
Main effect of long-term EE on fornix microstructure. Significant group differences were found for fornix fractional anisotropy, FA **(A)** and fornix mean diffusivity, MD **(B)**. **(A,B)** Significantly better white matter microstructure (higher FA, lower MD) in the fornix was detected for participants with higher long-term EE (EE**+**, orange) compared to participants with lower long-term EE (EE–, green). Split violin plots display data adjusted for covariates (age, gender, education, crystallized intelligence, socioeconomic status, diagnostic category, and scanner site for fornix microstructure). Boxplots display the median and interquartile range between the 1^st^ and 3^rd^ quartile. Individual data points display data adjusted for covariates. *p < 0.05. Key: EE, environmental enrichment; EE–, lower long-term EE; EE**+**, higher long-term EE; FA, fractional anisotropy; MD, mean diffusivity.

The association between long-term EE and WM microstructure, measured by FA and MD, in the pre-selected TOIs is shown in Table 2. As the selected LEQ items that were our measure of EE correlated with years of education (*r* = 0.34), crystallized intelligence (*r* = 0.22), and SES (*r* = 0.29), we corrected for these collinear influences in the statistical analysis procedure. Even after controlling for these co-variates in the main and exploratory analyses, EE still explained unique variance in the reported analyses, thus demonstrating the importance of EE. We found significant group differences for fornix FA (β = 0.117, *p* = 0.033) and fornix MD (β = −0.147, *p* = 0.015), after adjusting for co-variates that normally contribute to cognitive reserve and resilience and were correlated with EE in our sample. More specifically, participants who reported participating in higher long-term EE had significantly greater WM microstructure in the fornix, as indicated by higher FA and lower MD, compared to participants with lower long-term EE (Figure 1). There were no significant group differences in the other TOIs (all *p*’s > 0.1).

**TABLE 2:** Results for the multiple regression analyses between long-term EE and WM microstructure.

Dependent variable	B	SE B	Beta	*p*	Total R^2^ (adj.)
Fornix FA	0.013	0.006	0.117	0.033*	0.567 (0.527)
Fornix MD	−6.450e-05	2.631e-05	−0.147	0.015*	0.472 (0.423)
Parahippocampal cingulum FA	0.002	0.008	0.020	0.763	0.357 (0.297)
Parahippocampal cingulum MD	−8.055e-06	1.083e-05	−0.046	0.458	0.441 (0.389)
Uncinate fasciculus FA	−0.010	0.006	−0.103	0.116	0.372 (0.314)
Uncinate fasciculus MD	−8.976e-06	1.188e-05	−0.052	0.451	0.301 (0.236)

Long-term EE was included as a binary predictor, dummy coded with lower long-term EE = 0, higher long-term EE = 1. Models adjusted for age, gender, education, intelligence, SES, diagnostic category, and scanner site (for WM microstructure). Reference categories for categorical variables: lower EE group as the reference group for long-term EE. Female as the reference group for gender. Older adults as the reference group for the diagnostic category. Scanner site 1 as the reference group for the scanner site.

*p < 0.05.

Key: B, unstandardized coefficient; CI, confidence interval, SE, standard error; Beta, standardized coefficient; R2, explained variance; FA, fractional anisotropy; MD, mean diffusivity.

### 3.3. Exploratory path analysis

**FIGURE 2:**
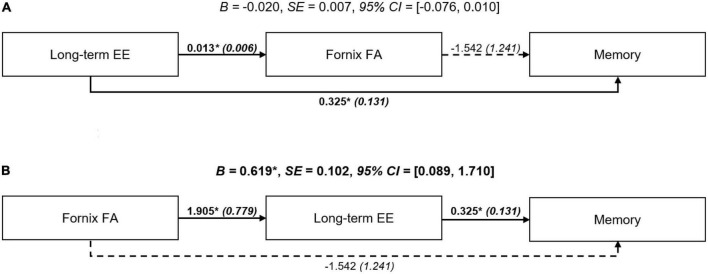
Path models investigating the association among long-term EE, fornix FA, and memory function. **(A)** Brain maintenance model: There was no statistical indication for a significant indirect (mediation) effect of the fornix FA. **(B)** Brain reserve model: Greater FA of the fornix was indirectly associated with better memory function through long-term EE. Path diagrams were adjusted for age, gender, education, crystallized intelligence, socioeconomic status, diagnostic category, and scanner site. Terms show unstandardized beta (B) coefficients and standardized errors (SE). Significant terms are indicated with bold font. Significant paths are indicated with continuous lines. Indirect effects with 95% CI are provided. *p < 0.05. Key: CI, confidence interval; EE, environmental enrichment; FA, fractional anisotropy.

Results of the exploratory path models are shown in Figure 2. The first path model (brain maintenance model) assessed long-term EE (lower/higher) as independent variable, fornix FA as mediator, and memory function as dependent variable. There was no indication that long-term EE was indirectly associated with memory function through fornix FA (unstandardized *B* = −0.020, SE = 0.007, bootstrapped bias-corrected 95% CI: −0.076, 0.010). A similar non-significant result was obtained for fornix MD (unstandardized *B* = −0.020, SE = 0.008, bootstrapped bias-corrected 95% CI: −0.081, 0.010, Supplementary Figure 2). The second alternative path model (brain reserve model) assessed fornix FA as independent variable, long-term EE (lower/higher) as mediator, and memory function as dependent variable. In this model, greater fornix FA was associated with better memory function through an indirect (mediation) effect of long-term EE (unstandardized *B* = 0.619, SE = 0.102, bootstrapped bias-corrected 95% CI: 0.089, 1.710), even after controlling for co-variates. A similar significant indirect effect was observed for the corresponding path model with fornix MD (unstandardized *B* = −0.035, SE = 0.005, bootstrapped bias-corrected 95% CI: −0.090, −0.010, Supplementary Figure 2).

### 3.4. *Post hoc* group characterization

We compared the two groups (lower/higher EE) to explore the relative differences in the frequency of participation across the 6 leisure activities (i.e., social, musical, artistic, physical, reading and additional language activities) to better characterize long-term EE. Compared to the lower EE group, participants with higher EE reported a greater frequency of participation in socio-cultural activities, resulting in large effect sizes of long-term EE (lower/higher) in musical (standardized regression coefficient: β = 0.538), artistic (β = 0.670), and additional language (β = 0.754) activities. In contrast, both EE groups (lower/higher) reported regular frequency of participation in the remaining leisure activities with small effect sizes of long-term EE in physical (β = 0.394), reading (β = 0.245), and social (β = 0.079) activities (Supplementary Table 1).

## 4. Discussion

### 4.1. Summary of findings

This study investigated the cross-sectional association between EE during life and WM microstructure of fiber tracts associated with the memory system in cognitively unimpaired OA of the DELCODE study. We compared two groups of OA that reported sustained higher or lower participation (between 13 and 65 years of age) in a variety of enriching leisure activities, adjusting for other known reserve proxies. We show that participants with higher long-term EE had better microstructure in the fornix tract (higher FA and lower MD) compared to participants with lower long-term EE. A similar effect was not seen in the other WM tracts. Follow-up exploratory path models suggested that greater fornix microstructure (higher FA and lower MD) was indirectly associated with better memory function through higher long-term EE. Our findings imply that sustained participation in a greater diversity of leisure activities may help preserve WM microstructure in the memory system of OA.

### 4.2. Enrichment and memory microstructure

We show that OA with higher long-term EE had more favorable microstructure in the fornix, both in terms of higher FA and lower MD, than OA with lower long-term EE. The present beneficial association between enriching leisure activities and fornix microstructure was found, accounting for other reserve proxies of education, crystallized intelligence, and SES (Stern, 2009; Jones et al., 2011) that were enhanced in the higher EE group. At the same time, we observed no significant differences between lower and higher EE for the other WM tracts, namely the uncinate fasciculus and the parahippocampal cingulum.

Our results align with and extend findings from previous review and meta-analytical studies reporting small and inconsistent effects of leisure activities on WM microstructure (Sexton et al., 2016; Anatürk et al., 2018; Duffner et al., 2023). One possible explanation for the selective sensitivity of the fornix, as observed in our study, may be that this major output tract of the hippocampus appears to be highly susceptible to aging processes (Zhuang et al., 2012; Hayek et al., 2020) and environmental challenges. The latter has been demonstrated by short-term learning/cognitive training studies in younger and older adults, respectively (Hofstetter et al., 2013; Antonenko et al., 2016). Intervention studies have further shown positive effects of longer-term music and dance trainings over 6 months on WM microstructure of the fornix in OA (Burzynska et al., 2017; Jünemann et al., 2022). In addition, other WM tracts seem to be important for the relation between EE and cognitive capacities. A longitudinal study in OA over 80 years showed that three year changes in WM microstructure in the corticospinal tract accounted for the association between changes in self-reported leisure activities and changes in perceptual speed (Köhncke et al., 2016).

Together, our and previous findings may reflect a lasting capability for neuroplastic changes or adaptations of WM microstructure including the fornix tract−even in late life−in response to or in interrelation with environmental challenges and experiences (see below). In light of this, our results may suggest that a greater variety of leisure activities could help preserve WM microstructure of the memory system in older age.

### 4.3. Exploratory path models

Path modeling showed a significant indirect association between fornix microstructure and late-life cognition through long-term EE. The result is challenging to interpret, given the cross-sectional nature of our study, which limits possibilities for causal inference. Nevertheless, our observation may suggest that greater fornix microstructure−if conceptualized as an indicator of brain reserve−could be associated with better memory function, when sustainably challenged by participation in enriching leisure activities. This interpretation aligns with the view that complex brain-behavior dynamics are associated with EE over the life course (Richards and Deary, 2005; Olszewska et al., 2021). It has been argued that more favorable brain properties in high-reserve individuals may facilitate exposure to or engagement in complex EE during life (see Maguire et al. (2000) and Olszewska et al. (2021) for a similar discussion). Other results have shown that a greater diversity of physically, intellectually and socially enriching activities in early life (before 13 years of age) are associated with variations in brain properties in later life (Morris et al., 2021). Greater brain resources (via predisposition or early EE) could support the maintenance of an enriched lifestyle throughout life and vice versa, which might promote a self-sustaining preservation of brain reserve into older age.

### 4.4. Enrichment and socio-cultural activities

We show that participants with higher long-term EE reported a more frequent participation in enriching leisure activities including music, art and language. Due to the multimodal sensory, motor, cognitive and social stimulation as inherent to socio-cultural activities (Wan and Schlaug, 2010; Herold et al., 2018), one could hence reason that participants in the higher EE group were more likely exposed to additive or synergistic effects of EE (Kempermann, 2019). Participants in the lower EE group also reported having performed enriching physical, cognitive and social leisure activities, albeit with relatively lesser frequency of participation in socio-cultural activities. The proposed benefits of leisure activities might thus be encouraged by the engagement in art, music, and other cultural activities, which have been associated with far-reaching positive effects on the brain and mental health (Fancourt and Finn, 2019). Regular participation in socio-cultural activities appears to act through broad neurobiological mechanisms to promote brain reserve and resilience in late life, which should be addressed in future studies.

### 4.5. Impact and significance

Taken together, our findings propose that long-term participation in a greater variety of leisure activities during young and middle adulthood may help preserve WM microstructure in the memory system of OA. This could be facilitated by the complex stimulation and integration processes associated with a physically, intellectually, and socially enriched lifestyle. In this line, studies have shown enhanced cognitive functioning after multimodal training compared with unimodal training in younger adults (Ward et al., 2017). The multimodal combination of modifiable lifestyle activities could serve as a low-threshold health strategy to foster brain and cognitive health throughout life to an older age. Targeted interventional studies with multimodal enrichment strategies will help to systematically evaluate the proposed merits of long-term EE. In addition, the relevance of socio-cultural activities in the benefits of an enriched lifestyle warrants further investigation.

### 4.6. Strengths and limitations

The present study indicates a positive association between self-reported participation in long-term EE during life and WM microstructure of fiber tracts associated with the memory system in a relatively large sample of clinically normal and well-characterized OA from the DZNE-DELCODE cohort (Jessen et al., 2018). To further investigate the sustained benefits of long-term EE, longitudinal studies with fine-tuned assessments of enriching leisure activities are needed to assess the progression of cognitive reserve and related brain properties over time. Several limitations need to be considered. (1) As the present results are based on cross-sectional data, caution needs to be taken concerning possible conclusions regarding the directionality of effects. Future longitudinal cohort studies could test the relation between environmental enrichment and fornix FA and MD over timespans to infer causality. (2) Self-reported retrospective measures of participation in leisure activities can be biased by the participant’s current cognitive status. However, the LEQ is considered an established instrument used in prior studies in cognitively unimpaired OA (Chan et al., 2018; Collins et al., 2021; Ourry et al., 2021) and we selected participants with a normal cognitive status from the DELCODE database. (3) The current study was translational in nature and applied group-based comparisons of lower and higher EE, to follow procedures from classical animal studies on enriched environments (Kempermann, 2019). Future studies may use other approaches to assess associations between EE, brain microstructure and memory and may additionally incorporate educational/occupational enrichment as assessed with the LEQ (Valenzuela and Sachdev, 2007). Further, it might be argued that exposure to earlier-life EE (before the age of 13 years) may facilitate later-life EE and brain functioning in older age (Morris et al., 2021). This assumption cannot be evaluated in the present study, as no information on early-life EE was available. (4) Studies with native diffusion tractography are needed to replicate the present results obtained with a probabilistic atlas-based tracts-of interest analysis. Prospective studies should explore other ways of addressing partial volume contamination from cerebrospinal fluid to see whether approaches with different methods yield robust results. It should be noted that head movements tend to increase with age, leading to more artifacts, as demonstrated in a resting-state study by Saccà et al. (2021). These head movements cannot always be entirely corrected through preprocessing and should therefore be considered as a possible limitation. (5) We did not find evidence of a direct cross-sectional association between fornix microstructure and memory performance in the present sample, although previous studies have reported such relations (Huang et al., 2007; Fellgiebel et al., 2008; Sexton et al., 2010; Hayek et al., 2020). Cross-sectional correlations between WM microstructure and cognitive function might be blurred by inter-subject heterogeneity in these measures, mixed brain pathologies and/or differences in reserve and resilience factors in our cohort.

## 5. Conclusion

Our results show that sustained participation in a greater diversity of leisure activities is associated with better fornix microstructure in OA. This beneficial association between long-term enrichment and brain reserve was found, accounting for other known reserve proxies such as SES, crystallized intelligence, and education. Regular engagement in multimodal physical, intellectual, and social enrichment during young and middle adulthood might represent an easily-accessible behavioral strategy to contribute to memory preservation and thus strengthen reserve mechanisms in late life.

## Data availability statement

The data analyzed in this study is subject to the following licenses/restrictions: the data of the DELCODE study are available on reasonable request (https://www.dzne.de/en/research/research-areas/clinical-research/databases-of-the-clinical-research/). Existing data analysis packages were used for statistical analyses. Respective scripts for the use of these packages are also available from the authors on reasonable request.

## Ethics statement

The general study protocol for the DELCODE study was approved by the Ethical Committees of the medical faculties of all sites, i.e., the Ethical Committees of Berlin (Charité – Universitätsmedizin), Bonn (Medical Faculty, University of Bonn), Cologne (Medical Faculty, University of Cologne), Göttingen (Universitätsmedizin Göttingen), Magdeburg (Medical Faculty, Otto-von-Guericke University, Magdeburg), Munich (Medical Faculty, Ludwig-Maximilians-Universität), Rostock (Medical Faculty, University of Rostock), and Tübingen (Medical Faculty, University of Tübingen). The process was led and coordinated by the Ethical Committee of the medical faculty of the University of Bonn under the registration number: 171/13. The patients/participants provided written informed consent to participate in the DELCODE study.

## Author contributions

AZ, MWa, SR, and MWi: conceptualization and design of the current study. MD, CB, KB, MB, PD, LD, ME, KF, SF, WG, SH, DJ, IK, LK, CL, FM, MM, RP, OP, JP, B-SR, NR, KS, ASc, ES, ASp, ST, JW, SW, RY, ED, FJ, MWa, and SR: overall design and implementation of the DELCODE study. ML, AZ, MG, DH, and MWi: methodology/statistical analysis. OK, AZ, and MWi: interpretation of data. All authors contributed to drafting and/or revision of manuscript and approved the final version.

## Footnotes

1
http://www.fil.ion.ucl.ac.uk/spm/

2
http://www.neuro.uni-jena.de/cat/
