# Supplementary material for: Long-term environmental enrichment is associated with better fornix microstructure in older adults
Source: Front Aging Neurosci. 2023 Aug 28;15:1170879. doi: 10.3389/fnagi.2023.1170879 (PMC10498282; doi:10.3389/fnagi.2023.1170879)
Supplement: Supplementary file 1 [file Data_Sheet_1.docx]

**Supplement**

**Long-term Environmental Enrichment is Associated With Better Fornix Microstructure in Older Adults**

# Supplementary methods

## Design of the DELCODE study and selection procedure

The DELCODE study aimed to recruit 1000 participants in total (aged ≥ 60 years), including diagnostic categories of older adults (OA), first-degree relatives of AD patients (family history, FH), as well as participants with subjective cognitive decline (SCD), mild cognitive impairment (MCI), and mild AD. Detailed descriptions for recruitment procedures, including inclusion and exclusion criteria, are available elsewhere (Jessen et al., 2018). At baseline, extensive neuropsychological, clinical, behavioral, and neuroimaging assessments were performed at each participating site. Assessments were carried out using Standard Operating Procedures (SOP) to minimize site-effects and ensure high-quality data. Quality assessment of the MRI images was performed by the DZNE Magdeburg.

The present sample of participants was selected from the DELCODE baseline database (data release for this study: 01.2021 with a total of n = 1079 participants) based on target group and data availability, as displayed in the **Supplementary Figure S1**.


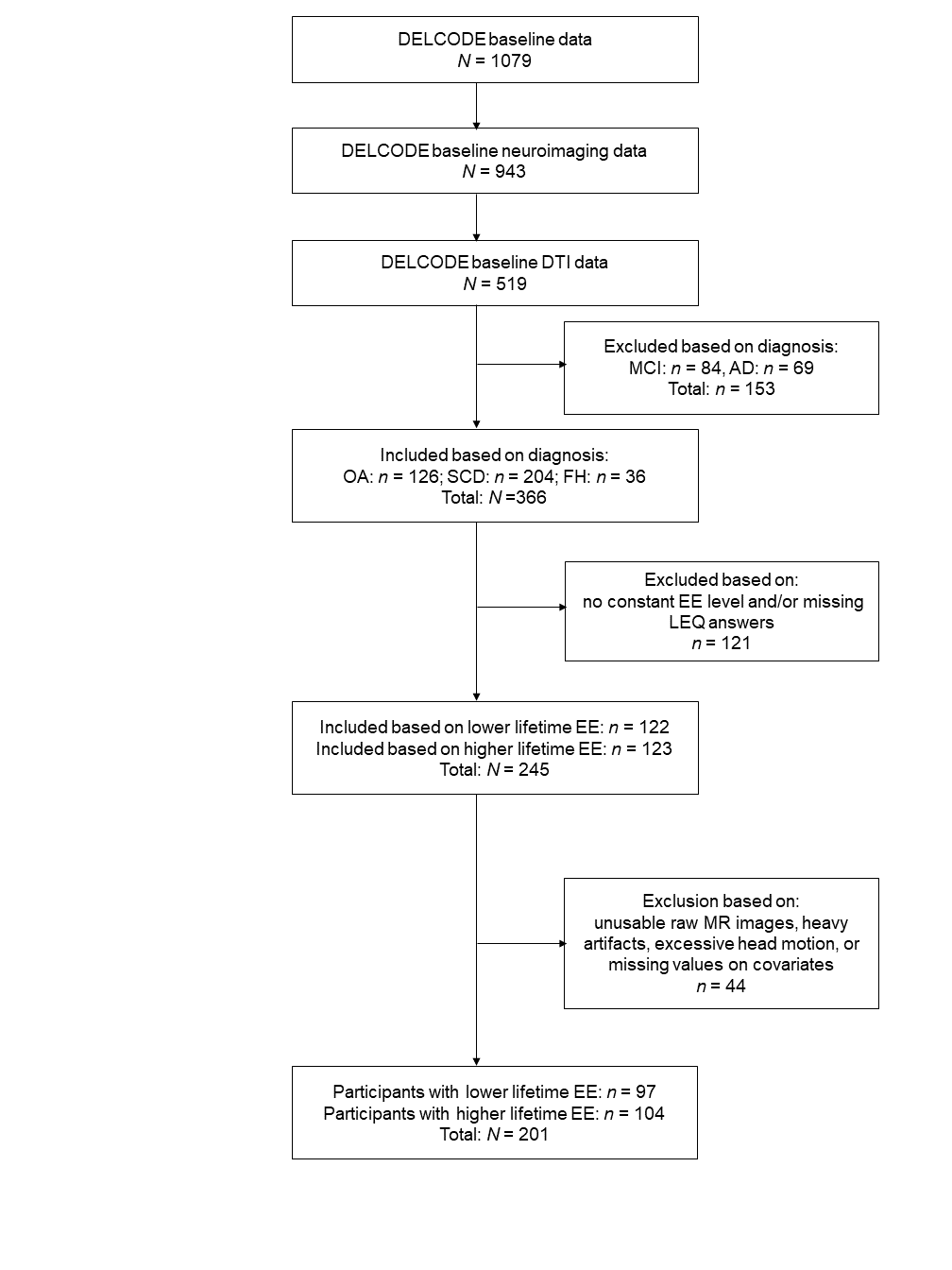


**Figure S1. Selection flow chart.** The baseline sample of the DELCODE study consisted at data release for the present study of N = 1079 participants. The present study considered participants with diffusion-weighted imaging data (n = 519). After exclusion of participants with MCI (n = 84) or AD (n = 69), the sample was reduced to n = 366 participants (OA, FH, SCD). Participants that did not fall into long-term EE (n = 77, defined as: lower/higher EE across younger adulthood and mid-life) and/or had missing LEQ responses (n = 44) were excluded (total: n = 121), reducing the sample size to n = 245. Lastly, participants that had unusable imaging data, heavy artifacts after preprocessing, excessive head motion or had missing values on covariates were excluded (n = 44). This resulted in a final sample of n = 201 participants, with n = 97 participants reporting lower long-term EE, and n = 104 participants reporting higher long-term EE. Key: OA: older adults, SCD: subjective cognitive decline, FH: family history, MCI: mild cognitive impairment, AD: Alzheimer’s disease, EE: environmental enrichment, LEQ: Lifetime of Experiences Questionnaire.

## Acquisition of MRI data

The MRI data were collected in nine participating sites, each equipped with a Siemens 3.0 Tesla MRI scanner (one Prisma, one Skyra, three TimTrio, and four Verio systems). T1-weighted anatomical images were obtained with a sagittal magnetization-prepared rapid gradient echo (MPRAGE) sequence (1 mm isotropic voxel size, field of view: 256 × 256 mm, matrix size: 256 × 256, echo time [TE]: 4.37 ms, repetition time [TR]: 2500 ms, flip angle: 7°, 192 slices, parallel imaging acceleration factor 2, duration: ~ 5min). The diffusion-weighted data were acquired with a single-shot echo-planar imaging sequence (2 mm isotropic voxel size, field of view. 240 × 240 mm, matrix size: 120 × 120, TE 88 ms, TR 12.100 ms, flip angle 90°, 10 b0 images, 60 gradients, i.e. 30 at b values 700 s/mm^2^ and 30 at b 1000 s/mm^2^, 72 slices, parallel imaging acceleration factor 2, ~ 15 min).

# Supplementary results

## Exploratory path analyses


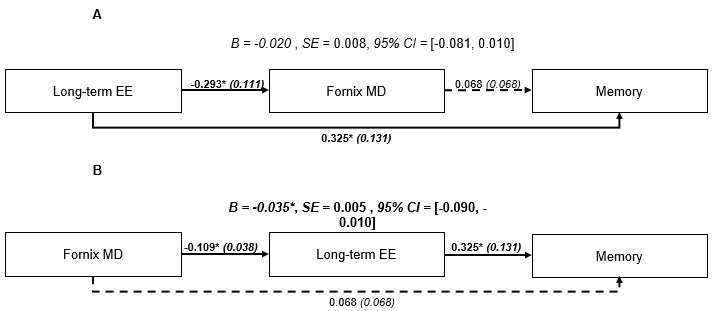


**Figure S2. Path models investigating the associations among long-term EE, fornix MD, and memory function.** **A.** Brain maintenance model: There was no statistical indication for a significant indirect (mediation) effect of the fornix MD. **B.** Brain reserve model: Lower MD of the fornix was indirectly associated with better memory function through long-term EE. Path diagrams were adjusted for age, gender, education, intelligence, SES, diagnostic category, and scanner site. Terms show unstandardized beta (B) coefficients and standardized errors (SE). Significant terms are indicated with bold font. Significant paths are indicated with continuous lines. Indirect effects with 95% CI are provided. * p < .05. **Key:** CI: confidence interval, EE: environmental enrichment; MD: mean diffusivity.

## Post-hoc group characterization

| ***Table S1.*** *Results of the post-hoc group characterization across the 6 leisure activities.* | | | | | |
| --- | --- | --- | --- | --- | --- |
| Dependent variable | Frequency of participation # | | B | SE B | Beta |
|  | Lower EE  M(SD) | Higher EE  M(SD) |  |  |  |
| Language | 0.371 (0.808) | 3.034 (1.420) | 2.663 | 0.165 | .754 |
| Artistic | 0.134 (0.379) | 2.284 (1.622) | 2.150 | 0.169 | .670 |
| Musical | 0.108 (0.403) | 1.640 (1.629) | 1.531 | 0.170 | .538 |
| Physical | 3.443 (1.325) | 4.322 (0.646) | 0.879 | 0.146 | .394 |
| Reading | 4.634 (0.840) | 4.938 (0.217) | 0.304 | 0.085 | .245 |
| Social | 4.479 (1.005) | 4.625 (0.847) | 0.146 | 0.131 | .079 |
| Long-term EE was included as a binary predictor, dummy coded with lower EE = 0, higher EE = 1. Reference categories for categorical variables: Lower EE group as the reference group for long-term EE.  # The mean frequency of participation in each leisure activity was measured using a 6-point Likert scale ranging from 0 (never) to 5 (daily) over early adulthood to mid-life. Means and standard deviations are reported for activities averaged over young adulthood and mid-life. Key: B: unstandardized coefficient; Beta: standardized coefficient; SD: standard deviation; SE: standard error; | | | | | |

# References

Jessen, F., Spottke, A., Boecker, H., Brosseron, F., Buerger, K., Catak, C., et al. (2018). Design and first baseline data of the DZNE multicenter observational study on predementia Alzheimer’s disease (DELCODE). Alzheimers. Res. Ther. 10, 15. doi: 10.1186/s13195-017-0314-2.
